# Supplementary material for: Oldhamite: a new link in upper mantle for C–O–S–Ca cycles and an indicator for planetary habitability
Source: Natl Sci Rev. 2023 Jun 5;10(10):nwad159. doi: 10.1093/nsr/nwad159 (PMC10476894; doi:10.1093/nsr/nwad159)
Supplement: nwad159_Supplemental_Files [file nwad159_supplemental_files.zip › Supplementary_for_NSR_MS-2022-1350.R2--2023.6.7.docx]

**Supplementary**

**1. Whole-rock and mineral composition of starting materials**

Calcium carbonate with a purity of 99.99% from the Alfa Aesar was used in this study. The orthopyroxenite samples were collected from the drillcore ZK11E05 at a depth of 259 m in the Xiarihamu giant Ni ore deposit from the Qinghai-Tibet Plateau (Fig. S1). The chemical composition of the orthopyroxenite samples is listed in Table S1. To some extent, the composition of orthopyroxenite can better reflect the environment of enstatite chondrite.


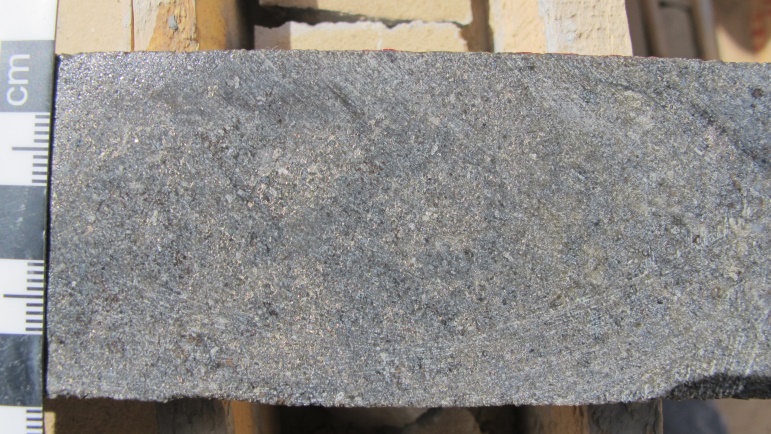


**Fig. S1. Core of orthopyroxenite at the depth of 259 m from drillcore ZK11E05 at the Xiarihamu giant Ni ore deposit in the Qinghai-Tibet Plateau.**

The orthopyroxenite is composed of 90% orthopyroxene and 5−7% clinopyroxene, with minor plagioclase (1-2%) and sulfides (Fig. S2A). The orthopyroxene grains in the rock are approximately 0.4 to 1.5 mm in diameter. The sulfides are primarily pyrrhotite and pentlandite (Fig. S2B) and are located mainly in the interstitial space between orthopyroxene grains. The composition of orthopyroxene is shown in Table S2.


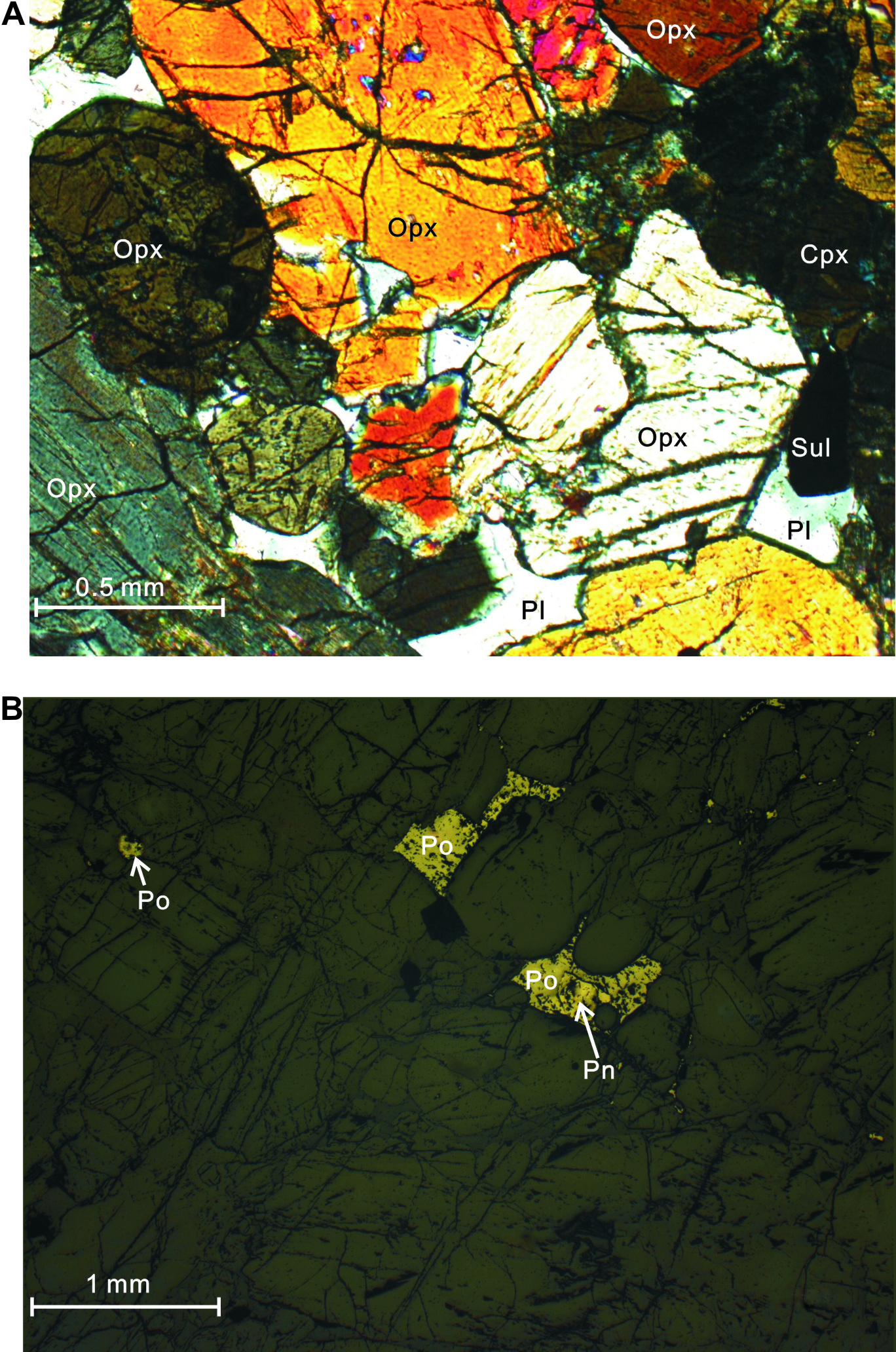


**Fig. S2. Photomicrographs of orthopyroxenite.**

(**A**) Photomicrograph of orthopyroxenite viewed under transmitted light.

(**B**) Photomicrograph of orthopyroxenite viewed under reflected light. Opx = orthopyroxene, Cpx = clinopyroxene, Pl = plagioclase, Sul = sulfide, Pn = pentlandite, and Po = pyrrhotite.

**2. Whole-rock major-element analysis**

Part of the orthopyroxenite sample was crushed in steel jaws to ~10 mesh powder, and ~200 g of initial powder was then further grounded to ~200 mesh using a tungsten carbide ring mill. Whole-rock Cu and Ni contents of the samples were measured via inductively coupled plasma atomic emission spectroscopy (ICP-AES), with a lower limit of detection of ~0.01 wt%, at ALS Chemex (Guangzhou) Co. Ltd. Whole-rock S contents were measured using a gravimetric method and IR absorption (IR08) by the infrared sensor at 1623 K with a lower limit of detection of ~0.01 wt. %. The analytical precision was ±8% of the amount present for S and ±3% for Ni and Cu. The other major element concentrations were determined using X-ray fluorescence (XRF) and a PANalytical Axios XRF instrument at ALS Chemex (Guangzhou) Co. Ltd. The results are presented in Table S1.

**3. High-pressure, high-temperature experiments**

Experiments at 0.5 GPa/1320 K and 1.5 GPa/1510 K were performed by a 2000-tons multi-anvil cubic apparatus at the University of Nevada, Las Vegas. We used semi-sintered MgO octahedra with 14 mm edge length as pressure-transmitting media coupled with pressure-generating cubic WC anvils with corner truncations of 8 mm edge length (Fig. S3B). The pressure was calibrated at room temperature using the cubic-orthorhombic transformation in PbS at 2.5 GPa [72], Bi II-III at 2.7 GPa [73], and Bi III-V at 7.7 GPa [74]. At 1473 K, pressure was calibrated using the quartz to coesite transformation at 3.2 GPa [75], a garnet/perovskite transition in CaGeO_3_ at 5.6 GPa [76], and the coesite to stishovite at 9.2 GPa [77]. The furnace assembly (14/8 “G2” box-heater assembly) consisted of a graphite sleeve and end-discs with moly electrodes at two ends, a zirconia outer sleeve, and a MgO inner sleeve.


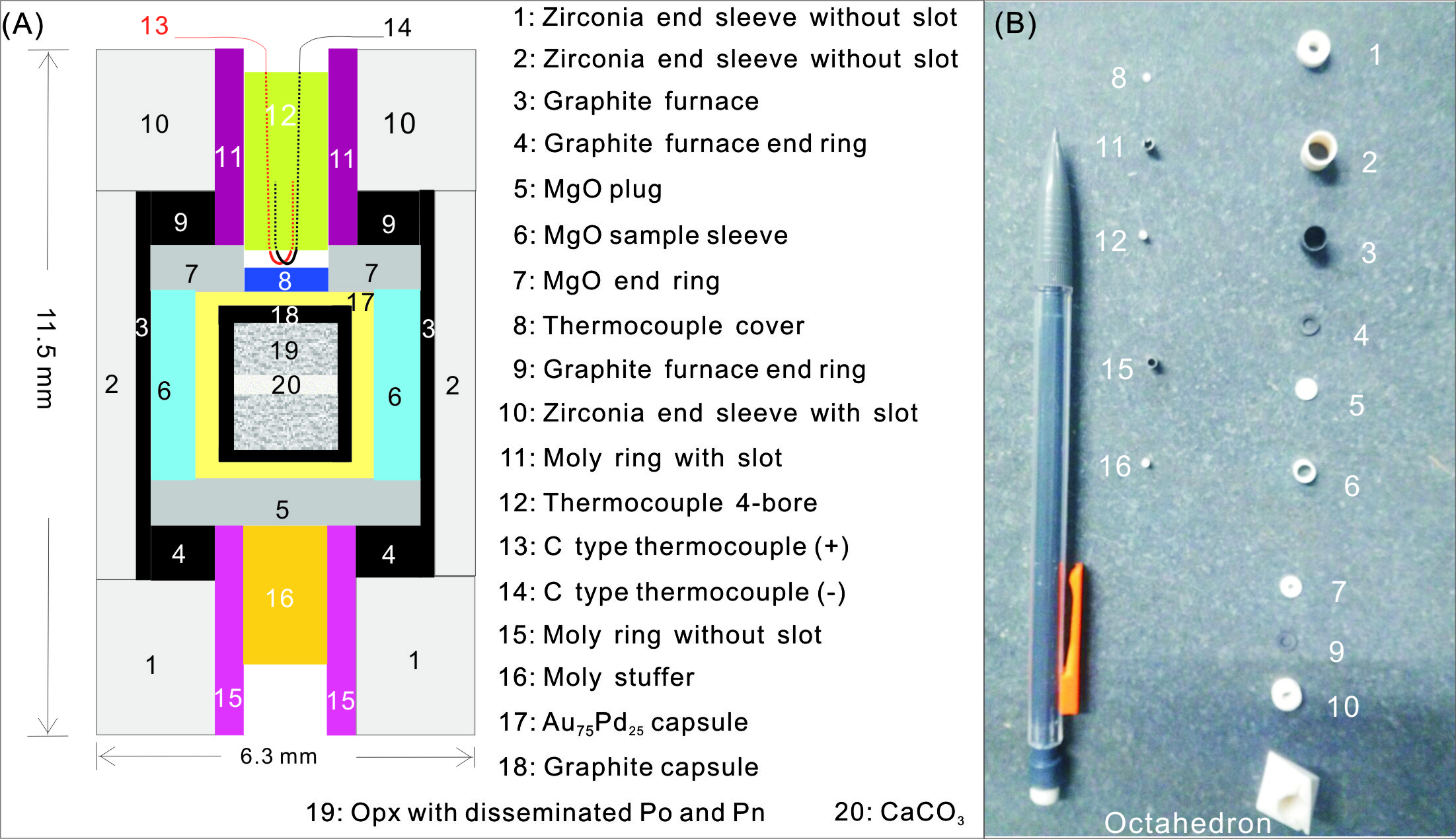


**Fig. S3**. Furnace assembly of multi-anvil cubic apparatus.

(**A**) Sketch map of 14/8 “G2” box-heater assembly for melting experiment. (**B**) The physical map of the main components in Figure A. Abbreviations: Opx = orthopyroxene, Po = [pyrrhotite](http://dict.youdao.com/w/pyrrhotite/#keyfrom=E2Ctranslation), and Pn = pentlandite.

Experiment at 0.3 GPa/1273 K was performed by a piston-cylinder press at the Institute of Deep-sea Science and Engineering, Chinese Academy of Sciences. The pressure medium is NaCl (Fig. S4). The temperature was measured with a C-type thermocouple (Fig. S4). The pressure was calibrated with the reaction of jadeite + quartz = albite [78] at 700 ºC for low temperature and low-pressure runs. At 1373 K, the pressure was calibrated using the quartz to coesite transformation at 3.2 GPa [75]. The pressure uncertainty at high temperatures was estimated as 6.2% at above 3 GPa and 29.6% at below 2 GPa, respectively.


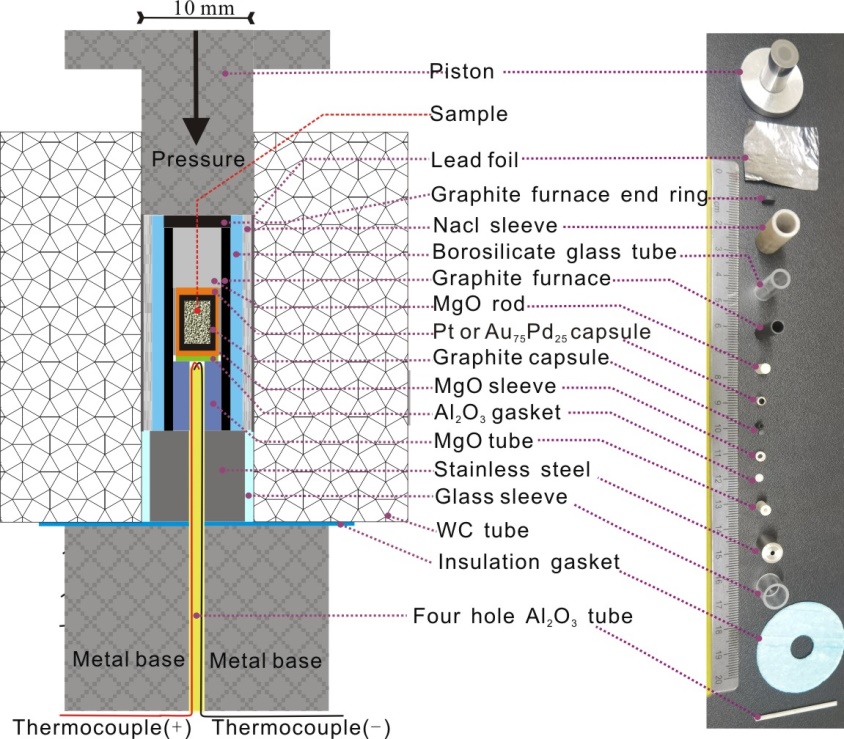


**Fig. S4**. Furnace assembly of piston-cylinder press.

.

In all experiments, sample powder was packed in a graphite crucible (3 mm in height, outer diameter = 2.7 mm, inner diameter = 2 mm) placed in an Au_75_Pd_25_ outer capsule with an outer diameter of 3 mm (Figs. S3A and S4). The assembled furnace parts and sample capsules were stored in a vacuum oven overnight at 533 K before each experiment. Contamination experiments No. C1, No. C2, and No.C3 between the pyrrhotite-pentlandite orthopyroxenite (PPO) and CaCO_3_ were performed at 0.5 GPa and 1320 K with a duration time of 1.2 hrs, at 1.5 GPa and 1510 K with a duration time of 1.2 hrs, and at 0.3 GPa and 1273 K with a duration time of 2 hrs, respectively. The amount of PPO and CaCO_3_ in each experiment was 70−79 mg and 10−16 mg, respectively. During the experiment, a sandwich structure was used, and calcium carbonate was placed in the middle (Fig. 1C and Fig. S3A). Before the contamination experiments, partial melting experiments of PPO powders at three *P-T* conditions (0.5 GPa and 1320 K for experiment No. P1; 1.5 GPa and 1510 K for experiment No. P2; 0.3 GPa and 1273 K for experiment No. P3) with a duration time of 1.2, 1.2, and 2 hrs, respectively, were performed. The silicate components of the melts produced by the partial melting of PPO are all basaltic melt. The results are shown in Table 3.

For the contamination experiments, the sample chamber after the contamination reaction can be divided into three reaction zones from the center to the edge, central reaction zone, marginal reaction zone, and partial melting zone (Fig. S5). The central reaction zone is characterized by the absence of silicate minerals and consists of melts, sulfides, or bubbles. The central reaction zone can be further divided into an inner part and an outer part. Bubbles and CaS exist only in the inner part (Figs. 1D and S5). Among the three contamination experiments, the reaction zone division from No. 1 (0.5 GPa/1320 K) is obvious. The melt composition of the inner part in this experiment (Melts-4 in Fig. S5 and Table S5) was featured by high CaO content (60.44−62.79 wt%), low MgO content (1.83−5.61 wt%), and high S content (Table S5). The CaO, MgO, and SiO_2_ content of the outer part melts (Melts-3 in Fig. S5 and Table S5) are 53.00−53.15 wt%, 6.34−15.11 wt%, and 28.51−29.68 wt%, respectively (Table S5). Compared with the inner part, the content of CaO and S in the outer part decreases significantly, the MgO content increases, and the SiO_2_ content does not change much. The marginal zone is characterized by an abundance of clinopyroxene (Fig. S5), which is formed by the reaction between orthopyroxene and high CaO-SiO_2_ melts (Opx + 2CaO+ 2SiO_2_ = 2Cpx). The composition of clinopyroxene and melt in the marginal reaction zone are listed in Table S5. The melt in the marginal reaction zone (Melts-2 in Fig. S5 and Table S5) consists mainly of SiO_2_ (41.16−43.45 wt%), CaO (38.79−39.79 wt%), MgO (12.99−13.40 wt%), and minor FeO and Al_2_O_3._ The area far away from the reaction area is the partial melting zone (Fig. S5), which is composed of residual orthopyroxene and basaltic melt (Melts-1 in Fig. S5 and Table S3).


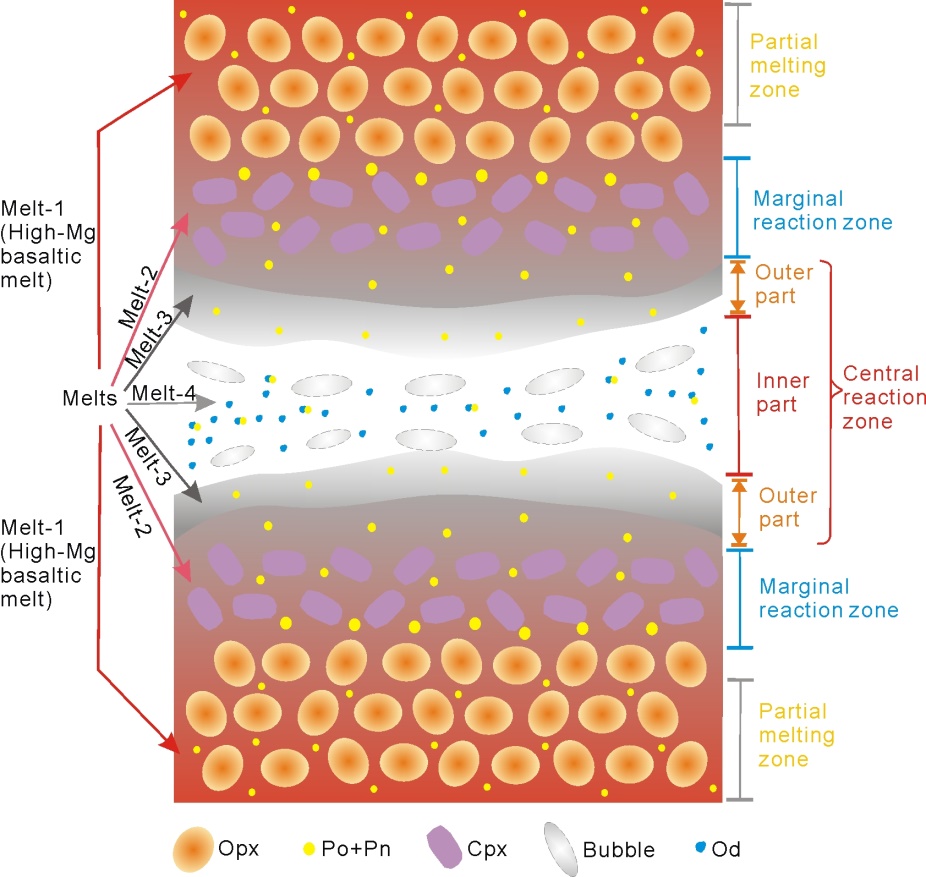


**Fig. S5. Typical reaction zoning of the sulfide-bearing magma-calcite (SMC).**

Abbreviations: Opx = orthopyroxene, Cpx = clinopyroxene, Po = [pyrrhotite](http://dict.youdao.com/w/pyrrhotite/#keyfrom=E2Ctranslation), Pn = pentlandite, and Od = oldhamite.

**4. Mineral and melt composition analyses**

The composition of minerals and melts from experiment No. P1, No. P2, No. P3, No. C1, and No. C2 was determined using an electron microprobe (instrument model JXA-8100) at Chang’an University. Analytical conditions included a voltage of 15 kV, a beam current of 20 nA, a beam diameter of 1−5 µm, and a peak-counting time of 20 s. The standard materials used in the analysis are as follows: albite, K-feldspar, hematite, forsterite, xenotime, corundum, wollastonite, Cr-spinel, kyanite, pyrophanite, barite, and metallic nickel. The detection limit for all elements was 0.01 wt% and the [relative error](http://www.so.com/link?url=http%3A%2F%2Fdict.youdao.com%2Fsearch%3Fq%3D%255B%25E5%2588%2586%25E5%258C%2596%255D%2520analytical%2520error%26keyfrom%3Dhao360&q=%E5%88%86%E6%9E%90%E8%AF%AF%E5%B7%AE+%E8%8B%B1%E6%96%87&ts=1499149739&t=617bc0ae95934046d656dcd206f1885) was ±2%.

The compositions of minerals and melts from experiment No. C3 were analyzed using an electron microprobe (instrument model JXA-iSP100) at the Guangzhou Tuoyan Testing Technology Co., Ltd with a beam diameter of 1−10 µm and a peak-counting time of 20 s. For sulfides, the voltage is 15 kV, and the beam current is 10−20 nA; for silicate minerals or melts, the voltage is 20 kV, and the beam current is 20 nA. The standards are the British MAC mineral/metal standard and the Chinese national standard sample GSB. For sulfides, the standard samples for Ca, S, Mg, Fe, and Ni are Co diopside (MAC), chalcopyrite (GSB), diopside (MAC), pyrite (GSB), and metal Ni (GSB), respectively. For silicate minerals or melts, the standard samples are albite (MAC), rutile (GSB), diopside (GSB), barite (GSB), apatite (GSB), magnetite (GSB), nickel oxide (GSB), corundum (MAC), orthoclase (MAC), manganese oxide (GSB), and chromium oxide (GSB). The detection limit for all elements was 0.01 wt%.

**5. Oxygen fugacity calculation**

**5.1. The determination of oxygen fugacity of Martian, Mercury’s, and lunar atmosphere**

The mean atmospheric pressure at the Martian surface is 0.061 bar [79], and the volume fraction of Martian oxygen is 0.782% (<https://www.engineeringtoolbox.com/>). Thus, the lg*f*o_2_ of the Martian atmosphere is –3.32.

The partial pressure of oxygen in Mercury’s atmosphere is about 2×10^−14^ bar [80]. Therefore, the oxygen fugacity of Mercury’s atmosphere lg*f*o_2_ is –13.7.

The upper limit for the density of oxygen atoms in the lunar atmosphere is 1.2×10^4^ cm^-3^ [81]. Thus, the upper limit of O_2_ density is 6×10^3^ cm^-3^. According to the ideal gas law, we can infer that the upper limit partial pressure of O_2_ in lunar atmosphere is 2.48×10^−16^ bar at 300 K. Therefore, the upper limit oxygen fugacity of lunar atmosphere lg*f*o_2_ is –15.6.

**5.2. The oxygen fugacity at the oldhamite–anhydrite equilibrium**

For the oldhamite–anhydrite equilibrium CaS_(s)_ + 2O_2_ = CaSO_4(s)_, at the standard state*P* = 1 bar and $T^{\circ}$ = 298.15 K, the standard Gibbs energy of formation for oldhamite and anhydrite is $\Delta_{f}G_{\mathrm{oldhamite}}^{\circ}$ = –469.5 kJ/mol and $\Delta_{f}G_{\mathrm{anhydrite}}^{\circ}$ = –1321.8 kJ/mol, respectively, and the molar volume *V_m_* for oldhamite and anhydrite is 27.72 cm^3^/mol and 46.01 cm^3^/mol, respectively [22].

At temperatures other than $T^{\circ}$, the Gibbs free energy of formation of the anhydrite $\Delta_{f}G_{\mathrm{anhydrite}}^{T}$can be calculated using the following regressed Equation S-1 [22]:

$\Delta_{f}G^{T}=A+BT+CT^{-2}$ (S-1)

where $A=-1.4465\times{10}^{3}$, $B=0.3971$, and $C=5.86\times{10}^{5}$. Note that Robie and Hemingway (1995) had an incorrect $A$ value for anhydrite, and we correct it here. We calculated the energy of formation for oldhamite at *T* ($\Delta_{f}G_{\mathrm{oldhamite}}^{T}$) by the following Equation S-2:

$\Delta_{f}G_{\mathrm{oldhamite}}^{T}= \Delta_{f}G_{\mathrm{oldhamite}}^{\circ}+\int_{T^{\circ}}^{T} C_{p}dT-T\int_{T^{\circ}}^{T} \frac{C_{p}}{T}dT$ (S-2)

where $\Delta_{f}G_{\mathrm{oldhamite}}^{\circ}$ is the standard Gibbs energy of formation (in kJ/mol) for oldhamite at the standard state*P* = 1 bar and $T^{\circ}$ = 298.15 K at equilibrium, and $C_{p}$ (J/mol/K) is the heat capacity.$C_{p}=A_{1}+A_{2}T+A_{3}T^{-2}$, where *A*_1_ = 46.29, *A*_2_ = 8.37 × 10^-3^, and *A*_3_ = –1.188 × 10^5^ [82]. At the equilibrium, we have the following Equation S-3:

$\Delta_{f}G_{\mathrm{anhydrite}}^{T}-\Delta_{f}G_{\mathrm{oldhamite}}^{T}+\left( P-P^{\circ} \right)\Delta V_{m}-2RT\ln fO_{2}=0$ (S-3)

where $P^{\circ}$ = 1 bar, *P* is the pressure (in bar) at equilibrium, *T* is the temperature in K at equilibrium, Δ*V*_m_ is the molar volume change of solid phases during the reaction, in cm^3^/mol, and R is the ideal gas constant 8.314 J/mol/K.

Thus, the oxygen fugacity at the oldhamite-anhydrite equilibrium is determined by the Equation S-4:

$$\lg fO_{2}=2.19144+1.09305\times{10}^{-4}T-\frac{25137}{T}-\frac{1551.42}{T^{2}}+$$

$1.5305\times{10}^{7}/T^{3}+0.04777P/T+2.7838\lg T$ (S-4)

where *P* is in bar, and *T* is in K.

**5.3. The oxygen fugacity at the oldhamite-lime-sulfur equilibrium**

For the oldhamite-lime**-**sulfur equilibrium 2CaS_(s)_ + O_2(g)_ = 2CaO_(s)_ + S_2(g)_, at the standard state*P* = 1 bar and $T^{\circ}$ = 298.15 K, the standard Gibbs energy of formation for oldhamite and lime is $\Delta_{f}G_{\mathrm{oldhamite}}^{\circ}$ = –469.5 kJ/mol and $\Delta_{f}G_{\mathrm{lime}}^{\circ}$ = –603.1 kJ/mol, respectively, and the molar volume *V_m_* for oldhamite and lime is 27.72 cm^3^/mol and 16.76 cm^3^/mol, respectively [22].

At temperatures other than $T^{\circ}$, the Gibbs free energy of formation of the lime $\Delta_{f}G_{\mathrm{lime}}^{T}$can be calculated using Formula 1, where $A=$–639.5, $B=0.1105$, and $C=3.497\times{10}^{5}$. The energy of formation for oldhamite at *T* ($\Delta_{f}G_{\mathrm{oldhamite}}^{T}$) was calculated by Equation S-2. At the equilibrium, we have the following Equation S-5:

$\Delta_{f}G_{\mathrm{lime}}^{T}-\Delta_{f}G_{\mathrm{oldhamite}}^{T}+\left( P-P^{\circ} \right)\Delta V_{m}-\frac{1}{2}RT\ln fO_{2}+\frac{1}{2}RT\ln fS_{2}=0$ (S-5)

where $P^{\circ}$ = 1 bar, *P* is the pressure (in bar) at equilibrium, *T* is the temperature (in K) at equilibrium, Δ*V*_m_ is the molar volume change of solid phases during the reaction, in cm^3^/mol, and R is the ideal gas constant 8.314 J/mol/K.

Thus, the oxygen fugacity at the oldhamite-lime**-**sulfur equilibrium is determined by Equation S-6 as follows:

$\lg fO_{2}=-21.1162+3.65342\times{10}^{7}\times T^{-3}-6205.07\times T^{-2}+(-16237.94-0.11450P)/T+0.43722\times{10}^{-3}T+11.13544 lgT+lgfS_{2}$ (S-6)

where *P* is in bar, and *T* is temperature in K.

**6.** **Calcium isotope fractionation between CaS and CaSO_4_**

The equilibrium Ca isotope fractionation between CaSO_4_ (Anh) and CaS (Od) can be determined by the following equations [18, 52]:

δ^44/40^Ca_Anh ­_– δ^44/40^Ca_Od_ =2.25×10^-5^ X^3^+4.01×10^-3^ X^2^+0.422X (S-7)

where X = 10^6^/*T* ^2^ , *T* is temperature in Kelvin. The temperature range for ﬁtting is 275–2275 K.

At 1543 K, the melt temperature of MORB [83], calcium isotope fractionation between CaSO_4_ (Anh) and CaS (Od) is δ^44/40^Ca_Anh_$-$δ^44/40^Ca_Od_ = 0.18‰.

**7. Possible area for the existence and evolution of CaS beneath a mid-ocean ridge**

Generally, at a depth of 160−170 km, the transformation from diamond to graphite occurs if the oxygen fugacity is above FMQ−2 (Fig. S6)[48]. At a depth of 150 ± 50 km [48], ‘redox melting’ [C (graphite) + 2Fe_2_O_3_ (melt) + O^2-^ (melt)= 4FeO + CO_3_^2-^ (both in the melt)] is considered to take place over a depth interval of 30 km, over which 30 ppm of carbon in the mantle source is oxidized (Fig. S6) [48, 84]. The ascending carbonate melt acts as a ﬂux to the overlying mantle and reacts with the mantle silicates to stabilize a carbonated silicate melt [49]. At a depth of about 60 km, the carbonate melt evolves toward a silicate melt composition (Fig. S6) [48]. The interaction between carbonate melt and sulfide-bearing magma could happen, providing conditions for the formation of oldhamite.


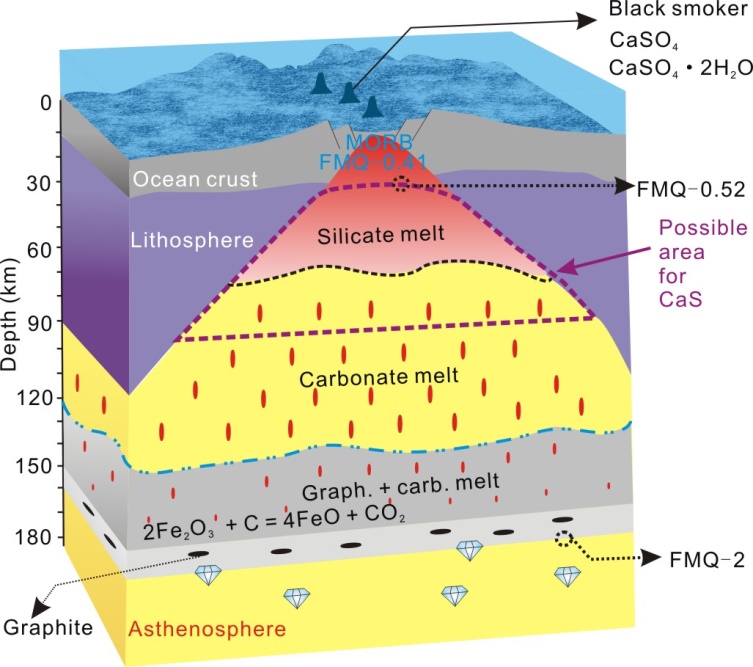


**Fig. S6. Possible area for the existence and evolution of CaS beneath a mid-ocean ridge.** The MORBs are characterized by a redox state of FMQ−0.41±0.43 (Fig. S6) [45], which is close to FMQ−0.52, the upper limit oxygen fugacity for the stable existence of oldhamite.

**8. The mass fraction of oldhamite in early Earth**

Early Earth was built by enstatite chondrites [60]. Based on the data in Table 1 and the fact that no oldhamite was found in some enstatite chondrites [85], it is difficult to determine the average mass fraction of oldhamite in enstatite chondrites (including EL and EH). That is, we cannot directly infer the mass fraction of oldhamite in early Earth from Table 1. But former researchers found out the sulfur concentration of early Earth accreted from enstatite-chondritic materials [86]. We assume that all the sulfur comes from sulfides. If we know the mass fraction of various sulfides in the enstatite chondrites, we can estimate the mass fraction of oldhamite in early Earth. But most studies about enstatite chondrites did not provide the mass fractions of all sulfides. Fortunately, the mass fractions of different sulfides in Khairpur (EL) and EET 87746 (EH) were reported [11, 13]. So we assume that the composition of Khairpur and EET 87746 represents the composition of EL and EH, respectively.

The sulfides in Khairpur (EL) are troilite (FeS_2_) and oldhamite (CaS), with a mass fraction of 7.03% and 0.34%, respectively [11]. Thus, the sulfur content in oldhamite accounts for 3.87 wt% ([0.34%×32/72]/[7.03%×64/120+0.34%×32/72]) of the total sulfur concentration in EL. Similarly, the sulfides in EET 87746, typical EH, are troilite, oldhamite, and niningerite (MgS), with a mass fraction of 14%, 3%, and 3%, respectively [13]. Thus, the contribution of sulfur in oldhamite to the total sulfur content of EH is 12.68 wt% ([3%×32/72]/[14%×64/120+3%×32/72+3%×32/56]). Early Earth composition corresponds roughly to a mixture of 1/3 of EL and 2/3 of EH [60]. Thus, the proportion of sulfur from oldhamites accounts for 9.74 % (= 3.87%×1/3+12.68%×2/3) of the total sulfur content of early Earth. The sulfur concentration of early Earth accreted from enstatite-chondritic materials is about 3,300−5,800 ppm [86]. Based on the above data, we can infer that the sulfur content from oldhamite in early Earth is 321.4−564.9 ppm. Then, the mass fraction of oldhamite in early Earth is estimated to be 0.072%−0.127% (from 321.4×72/32 ppm to 564.9×72/32 ppm).

Acknowledgments

We thank Professor Oliver Tschauner from the University of Nevada, Las Vegas for many in-depth discussions. We are very grateful to Professor D.L. Khosltedt from the University of Minnesota-Twin City and Professor Y.G. Xu and Dr. Z.Y. Luo from the Guangzhou Institute of Geochemistry, Chinese Academy of Sciences for their assistance in experimental petrology and sample preparation. Professor F. Huang and C. Zhou from the University of Science and Technology of China are acknowledged for their great help in isotope interpretation. Dr. Nanfei Cheng from the Institute of Deep-sea Science and Engineering, Chinese Academy of Sciences helped a lot during the experiment in the piston-cylinder press.

This work was supported by the Hainan Provincial Joint Project of Sanya Yazhou Bay Science and Technology City (2021CXLH0027 to Y.G.L.), the Central Guidance on Local Science and Technology Development Fund (ZY2021HN15 to S.H.M.), the Chinese Academy of Sciences (QYZDY-SSW-DQC029 and XDA22040501 to S.H.M.), the National Natural Science Foundation of China (No. 41973055 and No. 42130109 to I.M.C), and the Major Science and Technology Infrastructure Project of Material Genome Big-Science Facilities Platform supported by Municipal Development and Reform Commission of Shenzhen (L.P.W.)

Author contributions:

Conceptualization: Y.G.L., I.M.C., S.H.M. Methodology: Y.G.L., J.Z.C., L.P.W., N.P.W., W.Y.L., Z.R.L., and M.H.R. Investigation: Y.G.L., J.Z.C., N.P.W., and L.P.W. Visualization: Y.G.L., I.M.C., J.Z.C., W.Y.L., L.B., and M.H.R. Supervision: L.P.W., S.H.M., I.M.C. Writing—original draft: Y.G.L., J.Z.C., L.P.W., L.B., S.H.M., and I.M.C. Manuscript editing: S.H.M., I.M.C., L.P.W., L.B., Z.R.L., and M.H.R.

**Competing interests:** Authors declare that they have no competing interests.

**Data and materials availability:** All data are available in the main text or the supplementary materials.

Tables S1 to S5 (please see the separated .xlsx file)

**Table S1.** The major element concentrations of orthopyroxenite (wt%).

**Table S2.** The composition of orthopyroxene in orthopyroxenite.

**Table S3.** The melts composition during the partial melting experiments.

**Table S4.** Electron probe analysis results of typical oldhamite grains.

**Table S5.** Average composition of pyroxene (Cpx and Opx) and melts at different zones during the contamination experiments.

References in Supplementary

72. Zhou X, He D, Wang S*, et al.* New exploration on phase transition and structure of PbS under high pressure and temperature. *J Appl Phys* 2013; **113**: 043509.

73. Bundy F. Phase diagram of Bismuth to 130 000 kg/cm^2^, 500℃. *Phys Rev* 1958; **110**: 314–318.

74. Aoki K, Fujiwara S and Kusakabe M. Stability of the bcc structure of bismuth at high pressure. *J Phys Soc Jpn* 1982; **51**: 3826–3830.

75. Bose K and Ganguly J. Quartz-coesite transition revisited: Reversed experimental determination at 500-1200℃ and retrieved thermochemical properties. *Am Mineral* 1995; **80**: 231–238.

76. Ono S, Kikegawa T and Higo Y. In situ observation of a garnet/perovskite transition in CaGeO_3_. *Phys Chem Miner* 2011; **38**: 735–740.

77. Swamy V, Saxena S K, Sundman B*, et al.* A thermodynamic assessment of silica phase diagram. *J Geophys Res: Solid Earth* 1994; **99**: 11787–11794.

78. Holland T J. The reaction albite= jadeite+ quartz determined experimentally in the range 600–1200 ℃. *Am Mineral* 1980; **65**: 129–134.

79. Carr M H. The fluvial history of Mars. *Philos Trans R Soc* 2012; **370**: 2193–2215.

80. Broadfoot A L, Kumar S, Belton M*, et al.* Mercury's Atmosphere from Mariner 10: Preliminary Results. *Science* 1974; **185**: 166–169.

81. Halekas J, Poppe A, Delory G*, et al.* Using ARTEMIS pickup ion observations to place constraints on the lunar atmosphere. *J Geophys Res Planets* 2013; **118**: 81–88.

82. Petaev M and Khodakovsky I. Thermodynamic properties and conditions of formation of minerals in enstatite meteorite, in *Chemistry and Physics of Terrestrial Planets*: Springer, 1986, 106–135.

83. Morton J L and Sleep N H. A mid‐ocean ridge thermal model: Constraints on the volume of axial hydrothermal heat flux. *J Geophys Res: Solid Earth* 1985; **90**: 11345–11353.

84. Kadik A. Evolution of Earth's redox state during upwelling of carbon-bearing mantle. *Phys Earth Planet Inter* 1997; **100**: 157–166.

85. Udry A, Wilbur Z E, Rahib R R*, et al.* Reclassification of four aubrites as enstatite chondrite impact melts: Potential geochemical analogs for Mercury. *Meteorit Planet Sci* 2019; **54**: 785–810.

86. Wang W, Li C-H, Brodholt J P*, et al.* Sulfur isotopic signature of Earth established by planetesimal volatile evaporation. *Nat Geosci* 2021; **14**: 806–811.
